# Excess TPX2 Interferes with Microtubule Disassembly and Nuclei Reformation at Mitotic Exit

**DOI:** 10.3390/cells9020374

**Published:** 2020-02-06

**Authors:** Francesco D. Naso, Valentina Sterbini, Elena Crecca, Italia A. Asteriti, Alessandra D. Russo, Maria Giubettini, Enrico Cundari, Catherine Lindon, Alessandro Rosa, Giulia Guarguaglini

**Affiliations:** 1 Institute of Molecular Biology and Pathology, National Research Council of Italy, c/o Sapienza University of Rome, Via degli Apuli 4, 00185 Rome, Italy; francesco.naso@uniroma1.it (F.D.N.); v.sterbini@hsantalucia.it (V.S.); elena.crecca@uniroma1.it (E.C.); lia.asteriti@uniroma1.it (I.A.A.); alessandrarusso19@gmail.com (A.D.R.); enrico.cundari@uniroma1.it (E.C.); 2 CrestOptics S.p.A., Via di Torre Rossa 66, 00165 Rome, Italy; giubettini@crestopt.com; 3 Center for Life Nano Science, Istituto Italiano di Tecnologia, Viale Regina Elena 291, 00161 Rome, Italy; alessandro.rosa@uniroma1.it; 4 Department of Pharmacology, University of Cambridge, Tennis Court Road, Cambridge CB2 1PD, UK; acl34@cam.ac.uk; 5 Department of Biology and Biotechnology “C. Darwin”, Sapienza University of Rome, Piazzale Aldo Moro 5, 00185 Rome, Italy

**Keywords:** TPX2, mitosis, spindle, nuclear envelope

## Abstract

The microtubule-associated protein TPX2 is a key mitotic regulator that contributes through distinct pathways to spindle assembly. A well-characterised function of TPX2 is the activation, stabilisation and spindle localisation of the Aurora-A kinase. High levels of TPX2 are reported in tumours and the effects of its overexpression have been investigated in cancer cell lines, while little is known in non-transformed cells. Here we studied TPX2 overexpression in hTERT RPE-1 cells, using either the full length TPX2 or a truncated form unable to bind Aurora-A, to identify effects that are dependent—or independent—on its interaction with the kinase. We observe significant defects in mitotic spindle assembly and progression through mitosis that are more severe when overexpressed TPX2 is able to interact with Aurora-A. Furthermore, we describe a peculiar, and Aurora-A-interaction-independent, phenotype in telophase cells, with aberrantly stable microtubules interfering with nuclear reconstitution and the assembly of a continuous lamin B1 network, resulting in daughter cells displaying doughnut-shaped nuclei. Our results using non-transformed cells thus reveal a previously uncharacterised consequence of abnormally high TPX2 levels on the correct microtubule cytoskeleton remodelling and G1 nuclei reformation, at the mitosis-to-interphase transition.

## 1. Introduction

The assembly and disassembly of the mitotic spindle are tightly regulated and involve signalling cascades and the action of several microtubule (MT)-binding proteins, including MT nucleators, stabilising factors, motor proteins [1]. In this context, TPX2 (Targeting Protein for Xklp2) is emerging as a crucial element as it contributes in multiple ways to spindle assembly and function [2]. TPX2 is a MT-binding protein that appears at interphase nuclei in S and G2 phases, localises at spindle MTs in mitosis and is degraded by proteasome at mitotic exit [2]. Initially identified as the recruiting factor for the kinesin Xklp2 at MTs [3], TPX2 was subsequently classified as the first member of the Spindle Activating Factors (SAFs), regulated by the small GTPase Ran [4]. In in vitro experiments, TPX2 promotes MT stability and stimulates MT nucleation [5,6,7,8]; interestingly, recent cryo-EM data shed new light on TPX2 interaction with MTs, providing structural basis to the complexity of TPX2 effects on MTs [9]. TPX2 has also been described as an essential factor in augmin-mediated branching MT nucleation using *Xenopus* egg extracts [10,11]. The function of TPX2 in spindle assembly also involves the recruitment of specific factors to MTs: besides Xklp2, TPX2 binds Eg5 with its C-terminus, contributing to localise it to MTs and influencing its motor activity [12,13]. Moreover, the N-terminus binds the Aurora-A kinase and mediates its localisation at spindle MTs [14,15]. TPX2 binding also importantly contributes to Aurora-A kinase activation [16,17,18] and stability [19]. The first 43 amino acids of TPX2 have been described as the region required for Aurora-A binding [16] and deletions within this region have been previously shown to impair Aurora-A/TPX2 interaction and TPX2 regulation of Aurora-A [19,20]. 

Altogether, TPX2 diversified functions justify the observations that its RNA-interference (RNAi)-mediated inactivation in human cells strongly impairs bipolar spindle assembly and mitotic progression, arresting cells at the prometaphase stage [15,21,22]. Similar results were obtained in a mouse model, where lack of TPX2 induced early embryonic lethality and TPX2-deficient mouse embryonic fibroblasts transiently arrested in prometaphase with abnormally assembled spindles and less stable K-fibres, and eventually exited mitosis without chromosome segregation [23]. Experiments in human tumour cells showed that TPX2 overexpression also affects spindle assembly [21,24]. Several tumours overexpress TPX2 [2,25,26,27], often within signatures of mitotic genes, frequently including Aurora-A [25,28,29]. Therefore, cancer cell lines may already display deregulated levels of mitotic factors [30] and the actual effect of increased TPX2 levels on an unperturbed mitosis would be more precisely addressed using non-cancer cells.

In the present study, we analysed the consequences of TPX2 overexpression on the mitotic process in a non-transformed cellular background, discriminating their dependency on Aurora-A interaction. We do observe spindle assembly defects and impaired progression through mitosis. Unexpectedly, excess TPX2, independent of its ability to interact with Aurora-A, affected spindle disassembly and nuclear reformation at mitotic exit, resulting in doughnut-shaped nuclei and defective assembly of the lamin B1 network. These results link TPX2 overexpression to defective chromatin organisation and loss of nuclear envelope (NE) integrity and highlight the importance of controlling TPX2 levels at ana-telophase for a correct mitosis-to-interphase transition.

## 2. Materials and Methods

### 2.1. Plasmid Generation

The plasmids epB-Bsd-TT-VENUS and epB-Puro-TT-FLAG-TPX2 were generated by inserting the coding sequence of VENUS (excised from the pVENUS-N1_AURKA plasmid [31]), FLAG-TPX2 (amplified from the plasmid pEGFP-TPX2res [19] with the oligos BamHI_FLAG_TPX2_Fw: GGCGGATCCATGGACTACAAGGACGACGATGACAAGATGTCACAAGTTAAAAGCTC; and NotI_TPX2_Rv: CAGCGGCCGCTTAGCAGTGGAATCGAGTGG) into the BamHI and NotI sites of the enhanced piggyBac transposable vectors epB-Bsd-TT and epB-Puro-TT [32]. For generation of the epB-Puro-TT-FLAG-Δ43TPX2 plasmid, the insert FLAG-Δ43TPX2 was produced by PCR from the plasmid epB-Puro-TT-FLAG-TPX2 with the oligos BamHI_FLAG_TPX2Δ43_Fw (GGCGGATCCATGGACTACAAGGACGACGATGACAAGAAGTTACTGGGGAAGAATG) and NotI_TPX2_Rv. The FLAG-Δ43TPX2 sequence was then inserted into the BamHI and NotI sites of the enhanced piggyBac transposable vector epB-Puro-TT [32].

### 2.2. Generation of Stable Cell Lines

Stable transgenic hTERT RPE-1 cell lines were generated by transfection of a plasmid encoding the piggyBac transposase together with inducible vectors for expression of VENUS alone (epB-Bsd-TT-VENUS), FLAG-TPX2 full length/VENUS (epB-Puro-TT-FLAG-TPX2 and epB-Bsd-TT-VENUS), FLAG-Δ43TPX2/VENUS (epB-Puro-TT-FLAG-Δ43TPX2 and epB-Bsd-TT-VENUS) or FLAG-Δ43TPX2 alone (epB-Puro-TT-FLAG-Δ43TPX2 and epB-Bsd-TT). Transfection was performed using Lipofectamine LTX (Invitrogen, Carlsbad, CA, USA). Then, 48 h after transfection, selection with blasticidin-S hydrochloride and puromycin (both 9 μg/mL; Sigma-Aldrich, St Louis, MO, USA) was applied. Resistant cells were propagated as a pool, and expression of exogenous proteins after administration of 1 μg/mL doxycycline hyclate (dox, tetracycline analogue; Santa Cruz Biotechnology, Dallas, TX, USA) was verified.

### 2.3. Cell Cultures, Synchronisation Protocols and Treatments

The human hTERT RPE-1 epithelial cell line immortalised with hTERT (kind gift of Prof. Jonathon Pines) and the derived stable cell lines described above were grown at 37 °C and 5% CO_2_ in complete DMEM/F12 (Dulbecco’s Modified Eagle Medium F-12) supplemented with 10% tetracycline-free foetal bovine serum (FBS).

When indicated, cells were treated as follows: (a) 100 μM monastrol (Tocris, Bristol, UK) for 12 h to arrest cells in prometaphase; (b) 6 μM RO-3306 (Sigma-Aldrich) for 22 h to block entry into mitosis; (c) 0.5% FBS for 42 h to induce quiescence; (d) 2 mM thymidine (Sigma-Aldrich) for 24 h followed by 10 h release in thymidine-free medium and mechanic shake off, to collect and re-plate mitotic cells; (e) for Brefeldin A treatment (200 ng/mL; Sigma-Aldrich), cultures were presynchronised as in (d) and treated 10 h after thymidine release, for the following 12 h; (f) for cytochalasin B (cyto B) treatment (6,25 μM; Sigma-Aldrich), asynchronous cultures were treated for 24 h, 1 h after dox induction. Mock-treated cultures were incubated with 0.1% DMSO (a−b and e−f) or DMEM/F12 supplemented with 10% FBS (c–d).

For MT depolymerisation experiments, cells were incubated on ice in the presence of 10 μM nocodazole (Sigma-Aldrich) while control cultures were kept at 37 °C in 0.1% DMSO; after 15 min cultures were fixed and processed for immunofluorescence (IF) as indicated.

### 2.4. Immunofluorescence (IF)

Cells grown on coverslips were fixed as follows: (a) −20 °C methanol, 6 min, or (b) 3.7% formaldehyde/30 mM sucrose in PBS, 10 min at room temperature, followed by permeabilisation in PBS containing 0.1% TritonX-100, 5 min at room temperature. Blocking and incubations with both primary and fluorescently labelled secondary antibodies were performed at room temperature in PBS containing 0.05% Tween 20 and 3% BSA. Cells were counterstained with 4,6-diamidino-2-phenylindole (DAPI, 0.1 μg/mL; Sigma-Aldrich) and mounted using Vectashield (Vector Laboratories, Burlingame, CA, USA) or ProLong Glass Antifade Mountant (Invitrogen) for super-resolution. Primary antibodies were human anti-centromere serum (Antibodies Incorporated, Davisa, CA, USA; 1:20), chicken anti-α-tubulin (Abcam, Cambridge, UK, ab89984, 1:100), mouse anti-α-tubulin (Sigma-Aldrich, T5168, 2 μg/mL), mouse anti-acetylated-α-tubulin (Sigma-Aldrich, T6793, 2 μg/mL), mouse anti-α-tubulin-FITC (Sigma-Aldrich, F2168, 20 μg/mL), mouse anti-γ-tubulin (Sigma-Aldrich, T6557, 21.8 μg/mL), mouse anti-Aurora-A (BD Transduction Laboratories, San Jose, CA, USA, 610939, 0.5 μg/mL), rabbit anti-FLAG (Sigma-Aldrich, F7425, 2 μg/mL), rabbit anti-giantin (Abcam, ab24586, 1:500), rabbit anti-lamin B1 (Abcam, ab16048, 1 μg/mL), rabbit anti-pericentrin (Abcam, ab4448, 1 μg/mL), mouse anti-TPX2 (Abcam, ab32795, 4 μg/mL). Samples were analysed using a Nikon Eclipse 90i microscope (Nikon Instruments S.p.A., Campi Bisenzio Firenze, IT, USA) equipped with 20× (N.A. 0.5), 40× (N.A. 0.75) and 100× (oil immersion; N.A. 1.3) objectives and a Qicam Fast 1394 CCD camera (QImaging) or with an inverted microscope (Eclipse Ti, Nikon) using a 60× (oil immersion, N.A. 1.4) objective and a DS-Qi1Mc camera (Nikon). Images were acquired using NIS-Elements AR 3.2 (Nikon); elaboration and processing was performed using NIS-Elements HC 5.02 (Nikon) and Adobe Photoshop CS 8.0. Quantitative measures of geometric features were performed using NIS-Elements HC 5.02 (nd2 file format). Statistical analyses were performed with GraphPad; specific tests are indicated in Figure legends.

### 2.5. In Situ Proximity Ligation Assay (isPLA)

In situ proximity ligation assay (*is*PLA) was performed on cells grown on coverslips and fixed with formaldehyde (see previous section) using the Duolink PLA kit (DUO92007, Sigma-Aldrich) according to manufacturer’s instructions. The amplification time was 80 min and the primary antibodies pair to detect the interaction was mouse anti-Aurora-A (BD Transduction Laboratories, 610939, 0.5 μg/mL)/rabbit anti-FLAG (Sigma-Aldrich, F7425, 2 μg/mL). In the same reactions, IF staining of the spindle was performed using a chicken anti-α-tubulin antibody (Abcam, Ab89984, 1:100). DNA was stained with DAPI as above. For quantification of PLA fluorescence signals, images of mitotic cells were acquired using a 100× objective (oil immersion; N.A. 1.3), along the *z*-axis every 0.4 μm for a range of 8 μm. Signals were counted within the mitotic spindle region in the Maximum Intensity Projection images, using the “Spot detection” function of the NIS-Elements HC 5.02 (nd2 file format).

### 2.6. Western Blotting (WB)

Asynchronously growing cells or mitotic cells collected by shake off after monastrol treatment (see above) were lysed in RIPA buffer (50 mM Tris-HCl pH 8.0, 150 mM NaCl, 1% NP40, 1 mM EGTA, 1 mM EDTA, 0.25% sodium deoxycholate) supplemented with protease and phosphatase inhibitors (Roche Diagnostic, Mannheim, Germany). Proteins were resolved by 10% SDS PAGE and transferred on a nitrocellulose membrane (Protran BA83, GE Healthcare, Chicago, IL, USA) using a semi-dry system (Bio-Rad Laboratories S.r.l., Segrate, MI, Italy). About 40 μg of extract per lane was loaded. Blocking and antibody incubations were performed at room temperature in TBS containing 0.1% Tween 20 and 5% low fat milk for 1 h; for FLAG detection the primary antibody incubation was performed overnight at +4 °C. Antibodies were: goat anti-actin (Santa Cruz Biotechnology, I-19, 0.2 μg/mL), mouse anti-cyclin B1 (BD Biosciences, San Jose, USA, 554177, 2 μg/mL), rabbit anti-FLAG (Sigma-Aldrich, F7425, 0.8 μg/mL), mouse anti-GFP (Roche Diagnostic, ab290, 0.02 μg/mL) for VENUS detection, mouse anti-TPX2 (Abcam, ab32795, 4 μg/mL), rabbit anti-TPX2 (Novus Biologicals, Centennial, CO, USA, NB500-179, 1:500). HRP-conjugated secondary antibodies (Santa Cruz Biotechnology) were revealed using the ECL Western blotting analysis system or ECL Prime Western blotting detection reagent (both from GE Healthcare). Where indicated, a quantitative analysis on scanned films was performed using Adobe Photoshop 8.0.

### 2.7. Time Lapse Video Recording

Cells seeded in 4-well micro-slides (Ibitreat, 80426, Ibidi, Gräfelfing, Germany) were observed with an inverted microscope (Eclipse Ti, Nikon) using a 40× (Plan Fluor, N.A. 0.60, DIC) or a 60× (Plan Apo, N.A. 1.4, DIC) objective (Nikon). During the whole registration, cells were kept in a microscope incubator (Basic WJ, Okolab) at 37 °C in 5% CO_2_. DIC or fluorescence (SiR-DNA) images were acquired every 5 or 7 min over 48 h using a DS-Qi1Mc camera (Nikon) (experiments shown in Figure 3C), or over 4 h using the Clara camera (ANDOR technology) (experiments shown in Figure 5B), and the NIS-Elements AR 3.22 software (Nikon). The SiR-DNA (0.25 μM; Cytoskeleton, Inc., Denver, CO, USA) dye for DNA detection was added to thymidine presynchronised cells after 10 h from washout. Movie processing and analysis were performed with NIS-Elements HC 5.02 (Nikon).

### 2.8. Structured Illumination Microscopy (SIM)

The acquisition of images from the FLAG-TPX2^Δ43^ cell line not overexpressing VENUS (see Supplementary Figure S2) was performed through a Nikon Eclipse Ti equipped with: X-Light V2 spinning disk combined with a VCS (Video Confocal Super resolution) module (CrestOptics) based on structured illumination; LDI laser source (89 North); Prime BSI Scientific CMOS (sCMOS) camera with 6.5 µm pixels (Photometrics, Tucson, AZ, USA). Images were acquired using the Metamorph software version 7.10.2 (Molecular Devices, San Jose, CA, USA) and a 100× objective (PlanApo Lambda, oil immersion, N.A. 1.45) and sectioning the slice in Z with a step size of 0.1 μm.

In order to achieve super-resolution, raw data obtained by the VCS module were processed with a modified version of the joint Richardson-Lucy (jRL) algorithm [33,34,35], where the out-of-focus contribution of the signal was explicitly added in the image formation model used in the jRL algorithm, and evaluated as a pixel-wise linear “scaled subtraction” [36] of the raw signal. The obtained VCS super-resolved images were elaborated for 3D reconstruction using the Imaris 8.1.12 software (Oxford Instruments, Abingdon, UK).

## 3. Results

### 3.1. Characterisation of TPX2 Levels and Localisation in Non-Transformed Overexpressing Cell Lines

**Figure 1:**
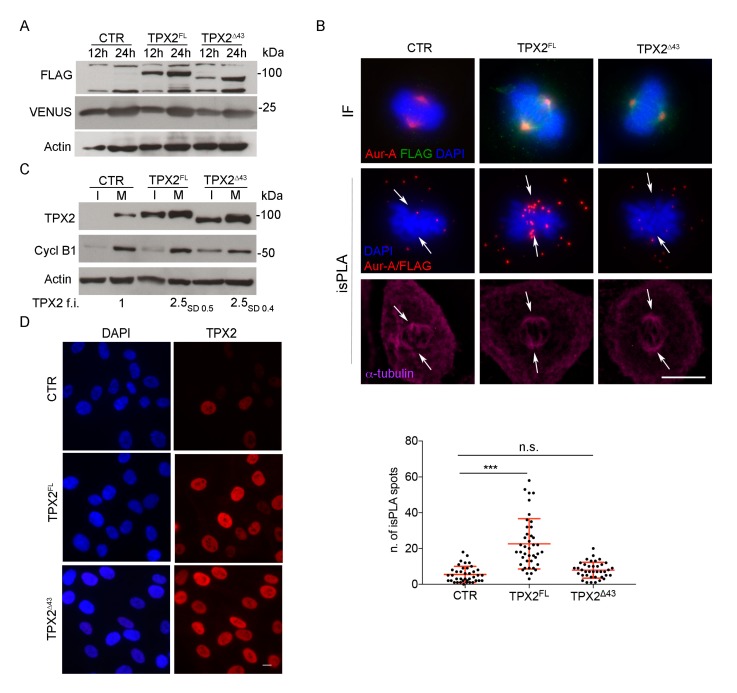
Generation of hTERT RPE-1 stable cell lines overexpressing TPX2 full length (FL) or Δ43. (**A**) Western blotting (WB) analysis shows exogenous TPX2 (FLAG-tagged) in the indicated cell lines, after 12 and 24 h of dox induction. Levels of the fluorescent VENUS protein are also shown; actin is used as loading control. (**B**) Upper immunofluorescence (IF) panels show the localisation of Aurora-A and FLAG-tagged proteins in mitosis in the indicated cell lines (the green signal comes from anti-FLAG staining, while VENUS fluorescence was lost due to methanol fixation); in situ proximity ligation assay (*is*PLA) signals corresponding to the FLAG/Aurora-A interaction are shown in the panels below. Arrows indicate the position of spindle poles, as assessed by α-tubulin staining. Quantification of the number of *is*PLA spots within the spindle area under the different conditions are also shown (measures from at least 40 mitoses from three independent experiments; standard deviations (s.d.) are shown; *** *p* < 0.0001, n.s. (not significant) *p* ≥ 0.05, one-way ANOVA, Tukey’s multiple comparisions test. (**C**) Total levels of TPX2 in the indicated cell lines in extracts from interphasic (I) and mitotic (M) cells, 12 h after induction with dox; cyclin B1 is used as a cell cycle control, actin as a loading control. Average fold increase (f.i.) of TPX2 levels in mitotic extracts is shown; subscripts indicate s.d. Molecular weights are indicated on the right side of the WB panels (**A**,**C**). (**D**) IF panels show interphase localisation and increased level of total TPX2 in the indicated cell lines after 12 h of dox induction. Scale bars: 10 μm.

In order to analyse the mitotic effects of TPX2 overexpression in non-transformed human cells, we generated hTERT RPE-1 cell lines for inducible expression of FLAG-TPX2 full length (TPX2^FL^) or FLAG-TPX2 Δ43 (TPX2**^Δ^**^43^), lacking the Aurora-A interaction region [16,20]; dox-dependent expression of exogenous TPX2 is not cell cycle regulated in this system, differently from the endogenous gene. Both cell lines, as well as the control (CTR) one, also expressed the VENUS fluorescent protein, as a read-out of the induction. The presence of the exogenous proteins after dox induction was assessed by Western blotting (WB) and IF analyses (Figure 1A and Supplementary Figure S1). To verify that, under our experimental conditions, overexpressed FLAG-tagged TPX2**^Δ^**^43^ does not interact with endogenous Aurora-A, while the FLAG-tagged TPX2^FL^ does, *is*PLA was used (Figure 1B): while both exogenous proteins colocalised with Aurora-A at spindle poles (upper IF panels), *is*PLA signals (in red, lower panels) were apparent and detected at poles only for the FL protein. Using an antibody directed against TPX2, which reveals both the endogenous and the exogenous protein, we determined a 2.5 fold increase of TPX2 levels (either FL or Δ43) after 12 h of dox induction in mitotic cell extracts (Figure 1C). A TPX2 antibody directed against aa 310–322 revealed a second faster migrating band in the TPX2**^Δ^**^43^ cell line (Supplementary Figure S2A,B), likely reflecting a further N-terminally deleted protein product; the same was observed with a second independently generated cell line, differing for lack of VENUS expression (Supplementary Figure S2C,D), suggesting that deletion of the first 43 amino acids of TPX2 unmasks a region within the N-terminus susceptible to cleavage.

**Figure 2:**
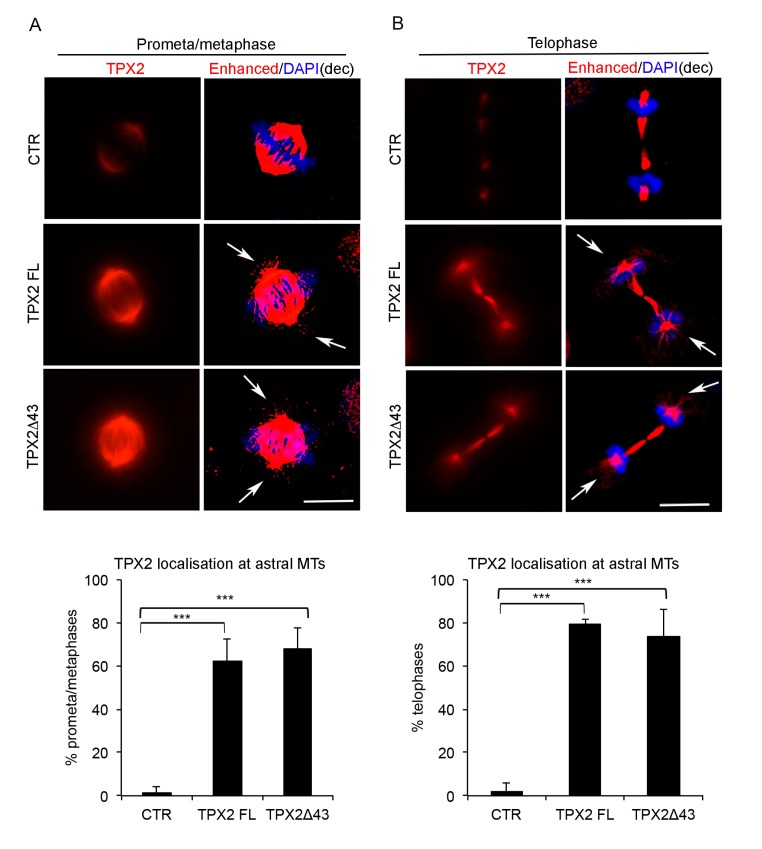
Mitotic localisation of TPX2 in overexpressing cell lines. Localisation of TPX2 at spindle microtubules (MTs) in VENUS (control—CTR) and TPX2-overexpressing cell lines in late prometa/metaphase (IF panels in (**A**)) or telophase (IF panels in (**B**)) cells (12 h induction, formaldehyde-fixed). Enhanced deconvolved panels are shown on the right column of each panel, to depict TPX2-positive astral MTs (white arrows) that are not present in the control cell line. Scale bars: 10 μm. Histograms below show the percentage of late prometa/metaphases (**A**) or telophases (**B**) displaying TPX2 staining at astral MTs. At least 50 cells per condition were counted, from three independent experiments. Bars indicate standard deviations. *** *p* < 0.0001, chi-square (and Fisher’s exact) test.

In both overexpressing cell lines (FL or Δ43), TPX2 correctly localises at spindle MTs (Figure 2); interestingly, a fraction of late prometaphases or metaphases displayed TPX2 staining at astral MTs (Figure 2A, IF panels and histograms), where TPX2 is normally excluded when expressed at endogenous levels (Figure 2A; [21]). IF analysis also showed that in telophase cells, when endogenous TPX2 starts decreasing through proteasome-dependent degradation [37], a strong TPX2 staining was still evident at the intercellular bridge in TPX2-overexpressing cultures (FL or Δ43) and TPX2-decorated astral MTs (arrowed) were present (Figure 2B). Consistently, in the overexpressing cell lines, TPX2 protein levels were high in interphase cells too (>70% TPX2-positive cells), where in most cases the endogenous protein is barely detectable (Figure 1C,D and Supplementary Figure S2B,C).

Thus, we concluded that the non-transformed cell lines we have generated are suitable to investigate the effects of TPX2 overexpression and their dependency on Aurora-A interaction.

### 3.2. Overexpression of TPX2 in Non-Transformed Cells Interferes with Mitotic Progression

**Figure 3:**
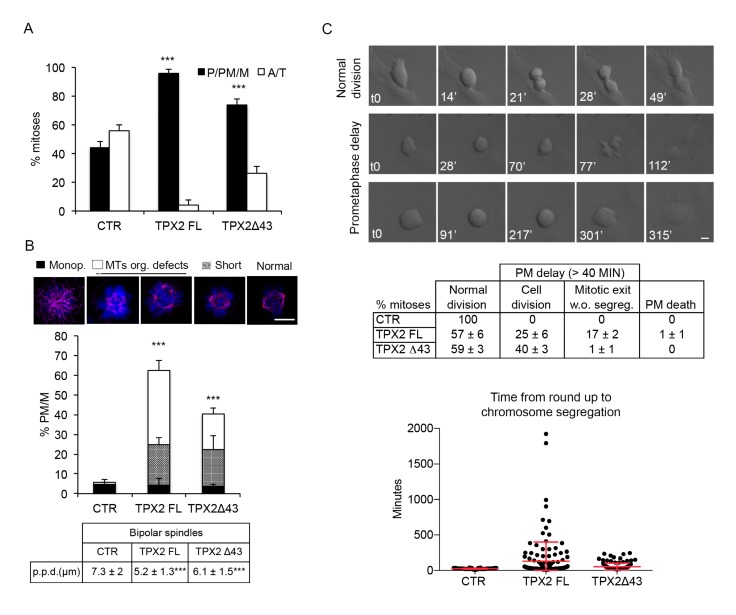
TPX2 overexpression delays progression through prometa/metaphase in non-transformed cells and yields cell division failure. (**A**) Histograms display the distribution in the indicated cell lines of mitotic cells in pro/prometa/metaphase (P/PM/M) or ana/telophase (A/T) after induction (24 h). A total of 300 mitotic cells were counted per condition, from three independent experiments; s.d. are shown. (**B**) TPX2 overexpression (24 h induction, methanol-fixed) leads to aberrant mitotic spindle organisation; quantification of scored defects (monopolar spindle, MT organisation defects, short spindles) exemplified in the IF panels (4,6-diamidino-2-phenylindole (DAPI) in blue; α-tubulin in purple), is shown in the histograms below. At least 250 prometa/metaphases (PM/M) were counted per condition, from three independent experiments; s.d. are shown. *** *p* < 0.0001, chi-square (and Fisher’s exact) test. The table below indicates the pole-to-pole distance (p.p.d.) measured in bipolar spindles only, in late prometaphases/metaphases under the indicated conditions. At least 90 spindles were measured per condition, from three independent experiments. *** *p* < 0.0001; one-way ANOVA, Tukey’s multiple comparisons test. (**C**) Time lapse video recording of VENUS (CTR) and TPX2 (FL or Δ43)-overexpressing cells, from the time of induction for the following 48 h. Differential interference contrast (DIC) still frames show a normal mitosis (top), and two examples of delayed prometaphase, either followed by cell division (middle) or by re-adhesion without chromosome segregation (lower panels). Minutes from round-up (t0) are indicated. Phenotypes (%) are shown in the table, while the dot plot indicates the time required (minutes) from round-up to the first signs of chromosome segregation (single measures, as well as the average values and s.d., are shown). Each dot represents a single mitosis; at least 100 cells were analysed per condition, from three independent experiments. Cells spending “>average control time + 2 s.d.” (>40 min) in a prometa/metaphase state were considered as delayed. Scale bars: 10 μm.

When we analysed TPX2^FL^ and TPX2**^Δ^**^43^ hTERT RPE-1 cell lines 24 h after dox induction, we noticed an increase in early mitotic figures (prometa- and metaphases) and a concomitant decrease in ana- and telophases (Figure 3A). A large fraction of prometa- and metaphases displayed spindle organisation defects, more frequently in FL- than in Δ43-overexpressing cells; spindles with reduced pole-to-pole distance (“short”) were also observed (Figure 3B). These observations suggest an arrest or strong delay in prometaphase; to investigate the actual fate of these mitotic cells, cultures were video recorded from the time of induction, for the following 48 h (Figure 3C). The average time spent by control cells from round-up to chromosome segregation was 25 min. This was significantly increased in TPX2-overexpressing cells, consistent with the high percentage of early mitotic figures observed in fixed samples: in both TPX2^FL^ and TPX2**^Δ^**^43^ cell lines, about 40% of dividing cells spent a significantly longer time in a prometa/metaphase-like state, with TPX2^FL^ cells being more dramatically delayed (average time 130 vs. 54 min for TPX2^Δ^^43^). Most TPX2**^Δ^**^43^-overexpressing mitoses displaying delayed prometa/metaphase eventually divided in an apparently normal bipolar manner (Figure 3C). In contrast, when TPX2^FL^ was overexpressed 17% of all cells entering mitoses showed prometaphase delay but subsequently failed cell division and re-adhered to the substrate without chromosome segregation; this behaviour was associated with a >150 min permanence in a prometaphase-like state, a condition rarely observed in TPX2**^Δ^**^43^ cells (Figure 3C). Thus, high levels of TPX2 interfere with normal progression through mitosis in non-transformed hTERT RPE-1 cells.

### 3.3. TPX2 Overexpression Affects the Intercellular Bridge in Telophase

**Figure 4:**
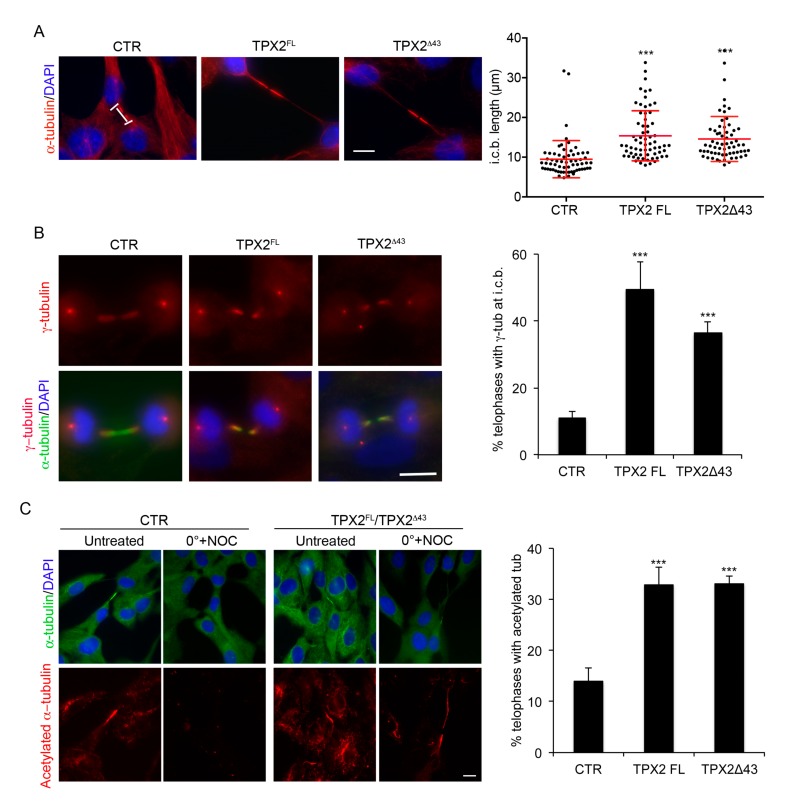
TPX2-overexpressing cells display abnormal intercellular bridges. (**A**) Intercellular bridge (i.c.b.) length was measured in late telophases (displaying decondensed chromatin) in the indicated cell lines; single measures, as well as the average value and s.d., are shown in the dot plot. About 65 telophases for each cell line were analysed, from three independent experiments. *** *p* < 0.0001; one-way ANOVA, Tukey’s multiple comparisons test. (**B**) Telophases displaying γ-tubulin at the i.c.b. were scored in the indicated cell lines; examples are shown in the IF panels. (**C**) Cells showing residual acetylated tubulin at the i.c.b. after treatment with nocodazole (NOC) on ice were counted, in the indicated cell lines; IF panels show the cultures under the different experimental conditions. About 300 telophases were analysed for each cell line, from three independent experiments; s.d. are shown. *** *p* < 0.0001, chi-square (and Fisher’s exact) test. In (**B**,**C**) the green signal comes from α-tubulin staining, while VENUS fluorescence was lost due to methanol fixation. Scale bars: 10 μm.

We then better characterised cells getting to ana-telophase in the presence of excess TPX2 (FL or Δ43; see Figure 2B). We first looked at the MT cytoskeleton in late mitotic figures (Figure 4). Intercellular bridges in TPX2-overexpressing cells displayed a significant increase in length compared to control telophases (Figure 4A), suggestive of increased stability and delayed disassembly. We, therefore, analysed whether nucleation or stabilisation of MTs (both TPX2-associated functions) following TPX2 overexpression were altered in telophase. We first assessed the localisation of γ-tubulin—the main MT nucleation molecule—at the intercellular bridge [38]: while only 11% of control telophases displayed γ-tubulin therein, this occurred in about 50% of TPX2^FL^, and 40% of TPX2**^Δ^**^43^, overexpressing telophases (Figure 4B), suggesting increased rates of MT nucleation. To assess whether MTs are also more stable than in control cells, we performed MT depolymerisation assays. Cells were placed on ice for 15 min in the presence of nocodazole and residual hyper-stable MTs were visualised using antibodies against acetylated tubulin. Under these conditions, only 13% control cells displayed acetylated tubulin at the intercellular bridge, while residual acetylated MTs were observed in 30% of TPX2-overexpressing telophases (both FL and Δ43; Figure 4C). Together, these data indicate that excess TPX2 at the end of mitosis leads to an abnormal frequency of telophases displaying γ-tubulin at the intercellular bridge and causes MT hyper-stabilisation, thus interfering with cytoskeleton disassembly.

### 3.4. Cells Overexpressing TPX2, Independently of Its Interaction with Aurora-A, Display a Doughnut-Shaped Organisation of Chromatin Starting from Telophase

**Figure 5:**
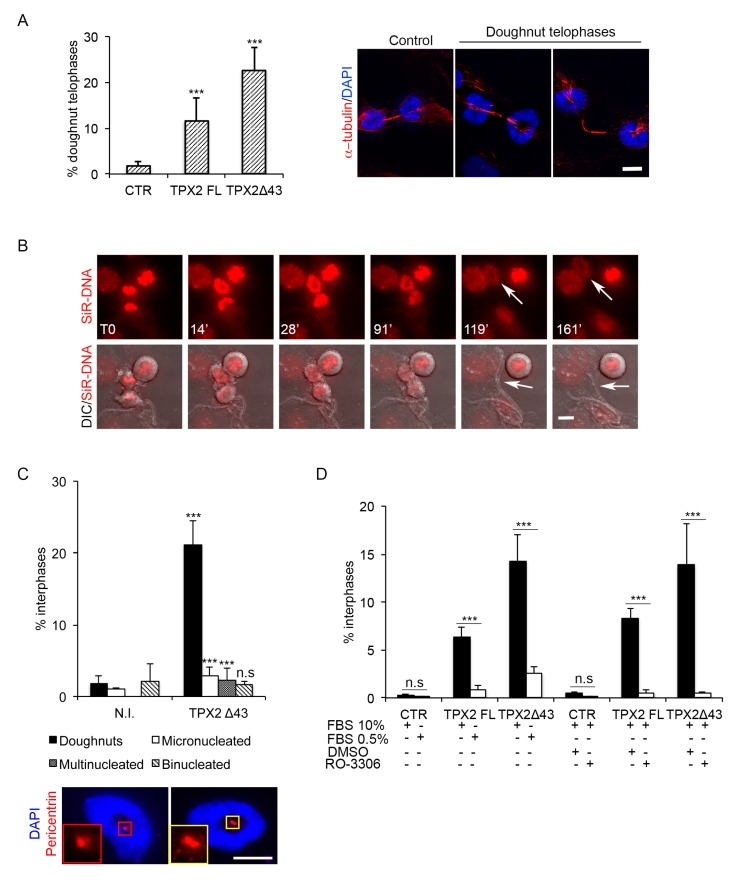
Doughnut-shaped nuclei formation requires passage through telophase. (**A**) Telophase cells with doughnut-shaped reforming nuclei, shown in the IF panels, appear in the indicated cell lines, as quantified in the histograms on the left (12 h induction). (**B**) Generation of doughnut-shaped nuclei in telophase was recorded by time lapse microscopy in the FLAG-TPX2^Δ43^ cell line not overexpressing VENUS (see Supplementary Figure S2); chromosomes are in red (SiR-DNA fluorescent label) and DIC images are merged in the lower panels. The first recorded telophase frame is indicated as T0. White arrows in the upper panels indicate the forming doughnut, while those in the lower panels indicate the intercellular bridge. (**C**) The percentage of defective interphases after mitotic shake off/re-plate in TPX2**^Δ^**^43^-overexpressing cultures (or non-induced controls, N.I.) is indicated in the histograms. The cell line with no VENUS expression (see Supplementary Figure S2) was used, in order to label centrosomes with anti-pericentrin antibodies, in addition to the DAPI and α-tubulin staining. The IF panels show examples of doughnut-shaped nuclei (DAPI) associated with one (left) or two (right) pericentrin spots (red); the insets show enlargements of the centrosomes. (**D**) Reduction of the doughnut-shaped nuclei phenotype was observed following serum starvation or RO-3306 treatment, in the indicated cell lines (+ and − indicate, respectively, the presence or absence of specific cell culturing conditions/treatments). Schematic representations of the experimental protocols are shown in Supplementary Figure S4. For all histograms, at least 1500 interphases per condition were analysed, from three independent experiments; s.d. are shown; *** *p* < 0.0001, n.s. (not significant) *p* ≥ 0.05, chi-square (and Fisher’s exact) test. Scale bars: 10 μm.

To investigate whether the abnormal MT assembly observed both in prometa-metaphases and telophases following TPX2 overexpression influenced chromosome segregation, we first analysed ana-telophases for the occurrence of mis-segregation events, such as chromosome bridges or lagging chromosomes (Supplementary Figure S3A). No increase of such events appeared when TPX2^FL^ or the truncated form were overexpressed. Interestingly, though, we noticed telophases with decondensing chromosomes displaying a peculiar doughnut-shaped distribution around astral MTs, which, in turn, appeared “connected” with MTs forming the intercellular bridge (Figure 5A). This phenotype was not dependent on the ability of overexpressed TPX2 to interact with Aurora-A, since it was observed both in TPX2^FL^ and TPX2**^Δ^**^43^ cell lines (about 10% and 20% doughnut-telophases, respectively; Figure 5A). Time lapse video recording using a chromosome fluorescent marker (SiR-DNA) confirmed the occurrence of doughnut-organised decondensing chromosomes in late telophases (Figure 5B, arrows in the fluorescent image), where an intercellular bridge is still present (Figure 5B, arrows in the DIC image). Persistence of this organisation in interphase was investigated by analysing a pure post-mitotic population: TPX2**^Δ^**^43^-overexpressing or control mitotic cells were harvested by mechanic shake off, re-plated and analysed after re-adhesion (post-mitotic cells; Figure 5C and Supplementary Figure S4A). A significant fraction of interphases displaying doughnut-shaped nuclei appeared under these conditions. Indeed, this was the most represented defect, accompanied by the presence of a small percentage of micro- and multi-nucleated cells; the few observed binucleated cells were present also in control cultures. Centrosomes, identified by pericentrin staining, were often positioned inside the doughnuts (Figure 5C, IF panels); the observation that doughnut-shaped nuclei were mostly (>70%) associated with a single centrosome indicated that these nuclei each belonged to a single daughter cell derived from an apparently normal cell division.

Consistent with these observations, doughnut-shaped nuclei were present in asynchronous cultures too, following overexpression of either TPX2^FL^ or TPX2**^Δ^**^43^, and were strongly reduced in number under conditions preventing cell division, i.e., serum starvation or impairment of mitotic entry by RO-3366 treatment (Figure 5D; Supplementary Figure S4B,C). Again, no significant induction of micronuclei and multinucleated cells was observed in the interphase population 24 h after induction (Supplementary Figure S3B). The observation that doughnut-shaped nuclei are consistently more represented in TPX2**^Δ^**^43^-overexpressing compared to TPX2^FL^-overexpressing cultures may reflect the fact that formation of such nuclei is dependent on the passage through telophase (which sometimes does not occur in TPX2^FL^-overexpressing mitoses; see Figure 3C) rather than on the TPX2/Aurora-A interaction. 

Overall, results so far show that cells dividing in the presence of excess TPX2 are defective for MT cytoskeleton disassembly and reformation of the interphase nucleus, in an Aurora-A-interaction-independent manner.

### 3.5. Lamin B1 Reassembly Is Defective in TPX2-Overexpressing Cells

**Figure 6:**
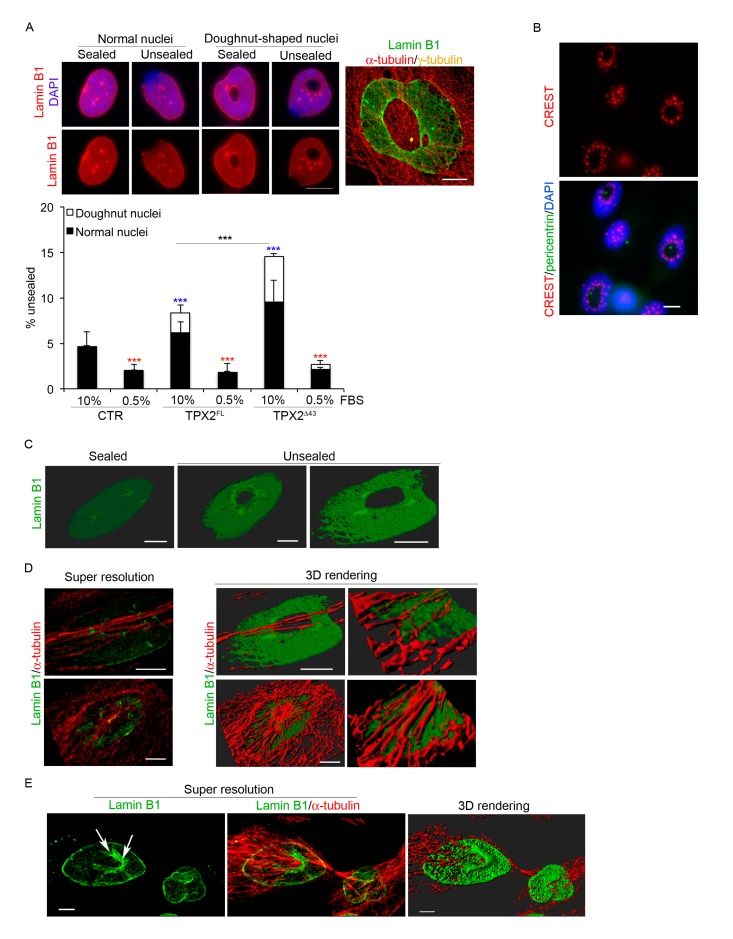
TPX2 overexpression interferes with lamin B1 organisation at the end of mitosis. (**A**) Overexpressing cells show a defective lamin B1 rim in normal and doughnut-shaped nuclei. The merged IF panel on the right is a super-resolved image exemplifying the defect. A total of 1500 cells were considered from three independent experiments for each cell line; s.d. are shown; *** *p* < 0.0001, chi-square (and Fisher’s exact) test. Blue asterisks refer to the difference from the control cell line, red ones to the difference, within each cell line, of serum starved cells (0.5% FBS) from control conditions (10% FBS). The statistical difference between TPX2^FL^ and TPX2**^Δ^**^43^ is also indicated. (**B**) Kinetochores (CREST, in red) organisation in doughnut-shaped nuclei is shown. The green signal comes from pericentrin staining, while VENUS fluorescence was lost due to methanol fixation. (**C**) Examples of 3D rendering of cell nuclei displaying sealed or unsealed lamin B1. (**D**) Examples of doughnut-shaped nuclei: left panels are volume views of super-resolved images, central ones are 3D rendering views; from the latter, enlargements of specific ROIs (Regions of Interest) are shown in right panels. (**E**) Volume view of a telophase cell with a doughnut-shaped nuclei forming, showing lamin B1 staining (left panel), merged with the α-tubulin signal (central panel). 3D rendering is shown on the right. Scale bars: 10 μm for widefield images and 5 μm for super-resolved images.

**Figure 7:**
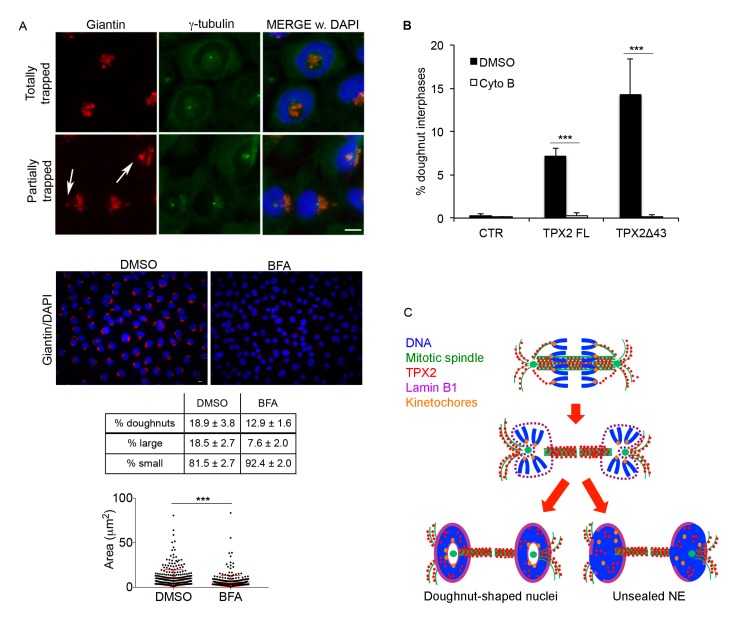
Golgi apparatus and cytoskeleton organisation contribute to doughnut-shaped nuclei generation. (**A**) Golgi organisation (giantin) in doughnut-shaped nuclei is shown in the upper IF panels. White arrows indicate trapped Golgi vesicles. Exemplifying images of control (DMSO) or Brefeldin A (BFA)-treated TPX2**^Δ^**^43^ overexpressing cultures are shown (red: giantin; blue: DAPI). The table indicates the percentage of doughnut-shaped nuclei in TPX2**^Δ^**^43^ cells after treatment with Brefeldin A (BFA; 1500 cells per condition were counted from three independent experiments); lower rows indicate the distribution of doughnut-shaped nuclei (at least 600 counted doughnuts per condition from 3–4 independent experiments) depending on the size of the hole. s.d. are indicated; *p* < 0.0001, chi-square (and Fisher’s exact) test for all BFA-treated vs. DMSO control cultures. Examples of “large holes” are in the upper IF panels, while “small” ones are exemplified in lower IF panels; “hole” measurements are shown in the graph below (300 measured doughnuts per condition, from three independent experiments; *** *p* < 0.0001; Mann–Whitney test). Scale bars: 10 μm. (**B**) Histograms indicate the percentage of doughnut-shaped nuclei in the indicated cell lines after cyto B treatment. A total of 1500 cells per condition were counted from three independent experiments; s.d. are shown; *** *p* < 0.0001, chi-square (and Fisher’s exact) test. (**C**) A model for the formation of doughnut-shaped nuclei and/or unsealed envelopes, from mitoses overexpressing TPX2, is depicted. The colour code is indicated. See text for details.

TPX2-overexpressing mitoses displayed abnormally disassembling MT cytoskeleton and aberrantly reforming nuclei at the mitosis-to-interphase transition. Considering the interplay between MTs and the reassembling nuclear envelope (NE) at this stage of the cell cycle [39,40], we investigated whether the abnormal MT stability due to excess TPX2 interferes with NE reformation. Cultures were stained with anti-lamin B1 antibodies (Figure 6A): first we observed that the inner part of doughnut-shaped nuclei was decorated by lamin B1, suggesting that NE reassembly occurs around decondensing chromosomes in the presence of persisting MTs. Interestingly, we noticed that chromosomal centromeric regions, as visualised by CREST staining, were mostly associated with this internal rim of lamin B1 (Figure 6B), supporting the idea that segregating chromosomes with pole-oriented kinetochores decondense around the persisting asters (see model in Figure 7C). In addition, while control nuclei displayed a continuous lamin B1 rim, a significant percentage of TPX2-overexpressing cells—particularly in cultures expressing TPX2**^Δ^**^43^—had an incompletely sealed envelope (Figure 6A), sometimes associated to DNA extrusions (“herniations”) corresponding to regions devoid of lamin B1. These defects were particularly evident when super-resolved images of doughnut-shaped nuclei were obtained, with large fenestrations becoming apparent (Figure 6A,C). The lamin B1 defect was not restricted to doughnut-shaped nuclei, but was present in apparently normal nuclei too, and was dependent on the proliferative state of the culture (Figure 6A, histograms), supporting the hypothesis that it occurs at NE reconstitution when cells exit mitosis. Super-resolved images of lamin B1 and MTs in both doughnut-shaped nuclei (Figure 6D) and late telophases (Figure 6E) also revealed MT arrays passing through the doughnuts hole and the lamin structure. The telophase image clearly shows lamin B1 closing around persisting MTs (Figure 6E, see arrowed detail) and extensive associations between MTs and lamins at a stage when contacts should be cleared for nuclear reformation [39,40]. These results suggest that the defect in cytoskeleton remodelling due to high levels of TPX2 (Figure 4) alters the integrity of the nuclear lamin and the shape of reforming nuclei at mitotic exit.

### 3.6. Reassembling Golgi Apparatus and Persisting MTs Contribute to Doughnut-Shaped Nuclei Generation

Altered lamin B1 assembly and NE resealing due to defective ESCRT-III/MT severing activity was previously described [40,41,42]. Still, under our conditions, this is also associated with a more general re-shape of nuclei reforming around MTs, suggesting that physical hindrance plays a major role. A potential cause of spatial hindrance could be represented by Golgi cisternae, which—at the mitosis-to-interphase transition—reassemble in the close proximity of centrosomes [43] often located inside the doughnuts (see Figure 5C and Figure 6B). We investigated Golgi distribution by staining cultures with anti-giantin antibodies (Figure 7A). Results indicate that Golgi cisternae were, totally or in part, trapped within the doughnuts, in association with centrosomes (Figure 7A). To explore the hypothesis that this may contribute to the peculiar shape of nuclei, we treated TPX2**^Δ^**^43^-overexpressing cultures exiting mitosis with Brefeldin A (see Supplementary Figure S4A), to impair Golgi reassembly in daughter cells. We show that Brefeldin A treatment yielded a completely disassembled Golgi and reduced both the formation of doughnut-shaped nuclei and the size of their holes (Figure 7A), suggesting that Golgi cisternae associated with the centrosomal aster contribute, through physical hindrance, to the formation of this peculiar nuclear shape. Still, the persistence of the defect suggests a further contribution of other cellular structures, in particular MTs. To investigate this issue and provide evidence that the persistence of hyper-stable MTs in telophase importantly contributes to defective nuclear reformation, we decided to specifically destabilise the cytoskeleton at the final stages of cell division. To this aim, instead of using MT-depolymerising agents that would strongly affect mitotic progression, we used cyto B, a drug that was shown to interfere with actin polymerisation and, as a consequence, with MT organisation at the cytokinesis stage [44]. While cyto B treatment altered cell morphology, it did not significantly affect mitotic index and the proportion of cells progressing through anaphase/early telophase; importantly, a significant proportion of cells exited mitosis, as testified by the presence of binucleated cells at the end of the treatment (Supplementary Figure S4D). Interestingly, under these experimental conditions, cyto B treatment totally abrogated doughnut-shaped nuclei formation (Figure 7B).

Overall, these results indicate that the mitosis-to-interphase transition and nucleus assembly are highly sensitive to TPX2 levels. Specifically, they show that TPX2 excess, by affecting the MT cytoskeleton in a manner that is independent of Aurora-A binding, strongly alters the reorganisation of nuclear structure and organelle distribution in daughter cells.

## 4. Discussion

In this work, we investigated the effects of increased levels of the MT-binding protein TPX2, frequently overexpressed in cancer, in non-transformed human cells. To address this question, we generated a cell line, starting from hTERT RPE-1 non-transformed cells, that overexpresses TPX2 in an inducible manner. A stable and inducible system was required since transient overexpression of TPX2 can lead to very high protein levels, causing MT bundling and cell cycle arrest or cell death [21,45]. Given the major role of TPX2 as an activator of the Aurora-A kinase, we generated a parallel cell line that overexpresses TPX2**^Δ^**^43^ (Aurora-A binding defective), to distinguish effects that are dependent or not on its ability to bind Aurora-A. Endogenous TPX2 levels and localisation are cell cycle-regulated [19,21]. Both parameters are affected in the TPX2-overexpressing cell lines: first, during early mitosis, TPX2 decorates astral MTs, only when overexpressed; second, rather than being degraded in telophase [37], overexpressed TPX2 persists at the intercellular bridge and astral MTs in telophase and is still observable in the majority of interphase nuclei. These observations indicate that constitutive, non-cell cycle-regulated TPX2 expression, as it may occur in cancer, leads to increased levels of protein and unbalances the physiological regulation of TPX2 during the cell cycle.

We report that TPX2 overexpression induces a strong delay of mitotic progression at the level of prometaphase, associated with aberrant spindle structure, as also shown in transformed cells [21,24,46]. This is, at least in part, independent of the TPX2/Aurora-A interaction, since mitotic delay, as well as spindle abnormalities, were observed in both the TPX2^FL^- and the TPX2**^Δ^**^43^-overexpressing cultures; still, TPX2^FL^ overexpression was significantly more efficient in inducing spindle organisation defects. This exacerbated phenotype is likely to cause the severe prometaphase delay observed by time lapse microscopy, which in half of the cases resulted in failed mitosis completion (mitotic slippage), following TPX2^FL^, but not TPX2**^Δ^**^43^, overexpression.

Interestingly, our approach enabled us to reveal a major effect of TPX2 overexpression at the mitosis-to-interphase transition, on the remodelling processes that involve the MT cytoskeleton and the reassembling NE, normally resulting in the reconstitution of a functional interphase nucleus [47,48]. In a significant proportion of TPX2-overexpressing post-mitotic cells, peculiar doughnut-shaped nuclei were observed, independently of TPX2 ability to interact with Aurora-A. A few examples of similar doughnut-shaped nuclei have been previously described following defective mitoses, associated with mitotic phenotypes including centrosome separation defects, centrosome amplification or chromosome mis-alignment/mis-segregation, thus possibly involving distinct generation mechanisms [49,50,51,52,53,54]; in some cases, their occurrence was associated with polyploidisation due to mitotic slippage, while, in our conditions, most cells bearing doughnut-shaped nuclei have undergone cell division. Indeed, this is likely the reason for TPX2**^Δ^**^43^-overexpressing cultures displaying a higher number of doughnut-shaped nuclei with respect to TPX2^FL^-overexpressing ones, where the occurrence of failed cell division is higher. Generation of doughnut-shaped nuclei in cells treated with protein farnesylation inhibitors [53], besides being associated with a centrosome separation defect and binucleated cells, was reported to require lamin B1, a farnesylation substrate. Interestingly, we show here that TPX2 overexpression, although not impairing centrosome separation, provokes defective reassembly of the lamin B1 network, suggesting a role for TPX2 regulation in the interplay between NE sealing and the control of MT dynamics. NE sealing has been proposed to be coordinated with MT disassembly through the action of MT-severing complexes at mitotic exit [40] and persistent MTs are reported to compromise nuclear morphology [42]. Super-resolved images enabled us to visualise a strongly compromised lamin B1 structure, interconnected to MTs, in TPX2 overexpressing cells. It is therefore possible that TPX2 excess influences nuclear reformation through its MT-regulating functions. Indeed, both centrosomal MT asters inside the hole of doughnut-shaped nuclei and the presence of stable and abnormally long intercellular bridges, suggest that MTs act as a physical hindrance for correct nuclear reformation when TPX2 is overexpressed (Figure 7C). The observation that destabilising the actin/MT cytoskeleton in late mitosis by cyto B treatment rescues the phenotype supports this hypothesis. However, we cannot exclude that actin, the cyto B primary target, contributes to the phenotype. Emerging evidence showing that TPX2 interacts with components of the actin-myosin network makes this possibility worthy of further investigation [55,56]. We show that the presence of reassembling Golgi cisternae, associated to the centrosome, inside the doughnut also contributes to the physical hindrance and abolishing its reassembly partially rescues the phenotype; whether Golgi trapping inside the doughnuts also results in its impaired functionality is an open question to be explored. MT-related effects of TPX2 overexpression appear largely independent of Aurora-A, since they were observed also after overexpression of TPX2**^Δ^**^43^, unable to bind the kinase. This would be consistent with recent data showing that TPX2, with augmin and the γ-TURC, are able to induce branched MT nucleation in vitro [57]. Still, the increased frequency of γ-tubulin-positive intercellular bridges recorded in TPX2-overexpressing cultures, as compared to controls, appears even higher in TPX2^FL^, compared to TPX2**^Δ^**^43^, overexpressing telophases, suggesting that in whole cells a contribution of Aurora-A to TPX2 MT nucleation functions exists. This would be consistent with the proposed role of Aurora-A in the nucleation of central spindle MTs [58,59]. Altogether, these observations support the hypothesis that an abnormally stable MT cytoskeleton in telophase interferes with nuclear reconstitution, leading to either defective reassembly of the lamin B1 network or, in extreme situations, to the closing up of the reforming nuclei around centrosomes and associated Golgi (Figure 7C). An additional, non-mutually exclusive, explanation for the effects of TPX2 excess on NE reformation may be represented by its ability to bind import receptors required for this process [60], leaving open the possibility of an unscheduled sequestration of the receptors at mitotic exit. Another intriguing possibility to be explored is the link, shown in the *Xenopus laevis* egg system, between TPX2 and lamina-associated polypeptide 2 (LAP2) proteins, proposed as an important interaction for post-mitotic nuclear reformation [61].

TPX2 overexpression is frequently observed in cancer [25,26,27] and high levels of TPX2 were shown to display the highest association score with chromosomally unstable tumours [28]. Still, under our conditions, we did not find obvious evidence of chromosome segregation errors in ana-telophases or micronuclei in the subsequent interphases. These data suggest that, although altering mitotic progression, TPX2 overexpression *per se* is not inducing a high percentage of chromosomal unbalanced daughter cells. Indeed, tumour-associated signatures, including TPX2 up-regulation, display overexpression of other cell cycle or mitotic regulators [24,28,29], and particularly of Aurora-A [25], suggesting that co-deregulation of other factors may underlie chromosomal instability in TPX2-overexpressing cells. In addition, TPX2 may have a role in the maintenance, rather than the establishment, of chromosomal instability in cancer cells, by fuelling mitotic errors through impairing correct spindle assembly.

Whether the doughnut-shaped nuclei and unsealed nuclear envelopes are present in TPX2-overexpressing cancers and constitute non-canonical routes to genome rearrangements leading to transformation is worthy of further investigation. Chromatin territories, which determine gene expression patterns and need to be re-established after mitosis [62,63], may be abnormally organised in those cells, since they depend on association with lamin B1 that, under our conditions, is both discontinuously distributed and abnormally lined around the central hole. Strikingly, CREST staining revealed a peculiar distribution of centromeric regions around the internal lamin B1 rim. A condition of discontinuous lamin B1 was also reported, following its RNAi-mediated inactivation, to be linked to defective DNA replication and repair [64]. Furthermore, the incomplete sealing of the NE may yield exposure of DNA to the cytoplasm, possibly triggering chromosomal rearrangements [65,66].

In conclusion, our results reveal the importance of lowering TPX2 levels in telophase for correctly regulating MTs and nuclear reformation, as well as for chromosomes and organelles reorganisation at mitotic exit, and opens the possibility of alternative routes, rather than canonical chromosome mis-segregation, for the transforming potential of TPX2.

## Supplementary Materials

The following are available online at https://www.mdpi.com/2073-4409/9/2/374/s1, Figure S1: Inducible overexpression of TPX2 full length (FL) or Δ43 in hTERT RPE-1 stable cell lines, Figure S2: TPX2Δ43 overexpression yields a further deleted N-terminal truncation, Figure S3: Chromosome segregation defects in TPX2-overexpressing cells, Figure S4: Experimental protocols for synchronisation in different phases of the cell cycle and drug administration.
