# Supplementary material for: Excess TPX2 Interferes with Microtubule Disassembly and Nuclei Reformation at Mitotic Exit
Source: Cells. 2020 Feb 6;9(2):374. doi: 10.3390/cells9020374 (PMC7072206; doi:10.3390/cells9020374)
Supplement: Supplementary file 1 [file cells-09-00374-s001.pdf]

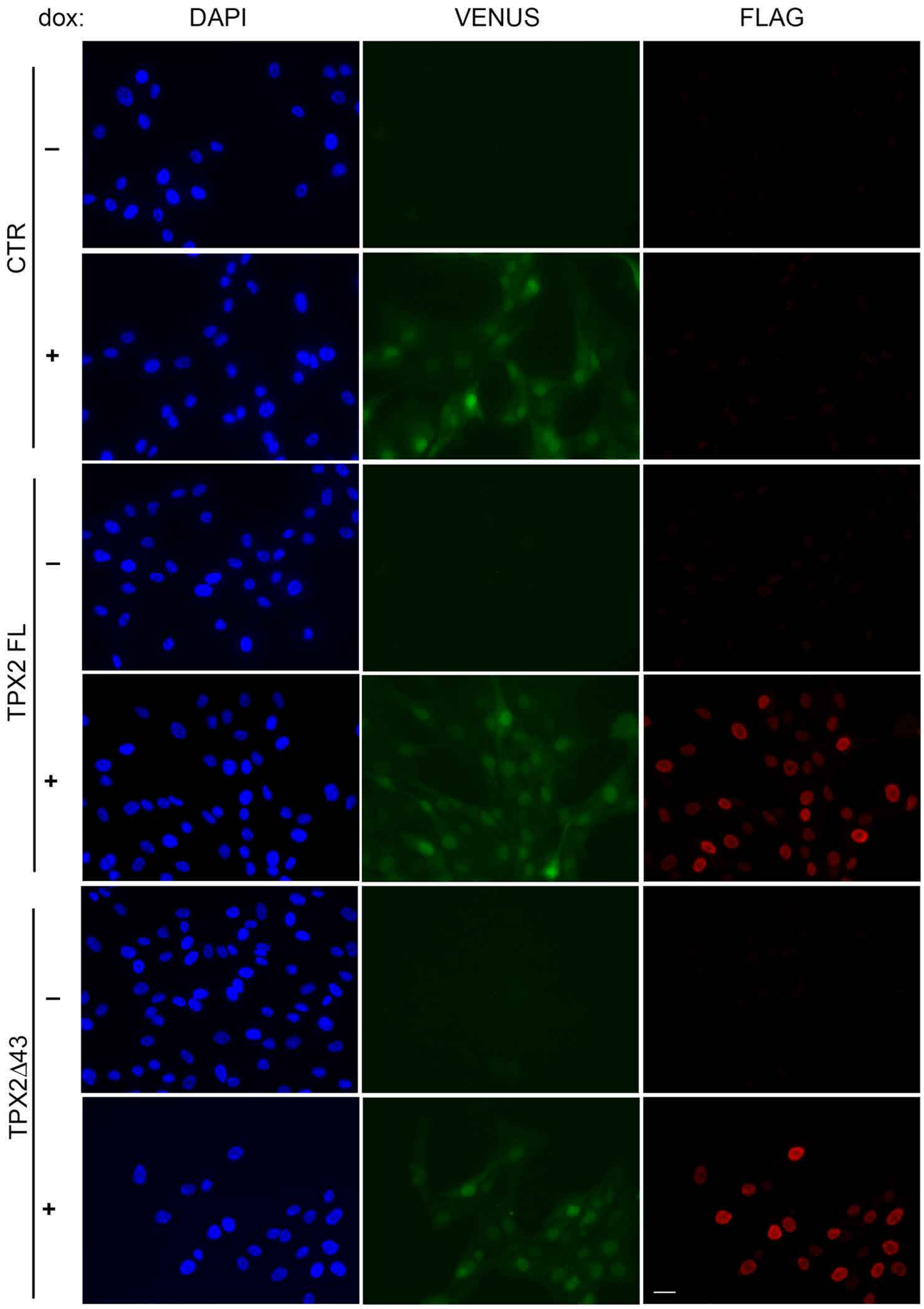

Figure. S1. Inducible overexpression of TPX2 full length (FL) or Δ43 in hTERT RPE-1 stable cell lines. Immunofluorescence panels show the inducible expression (+ dox) of FLAG-TPX2^{FL}, FLAG-TPX2$^{\Delta 43}$ and VENUS in the indicated cell lines after the induction (12 hours). Exogenous TPX2 displays the canonical localisation in interphase nuclei (visualised by DAPI staining). Scale bar: 25 μm.

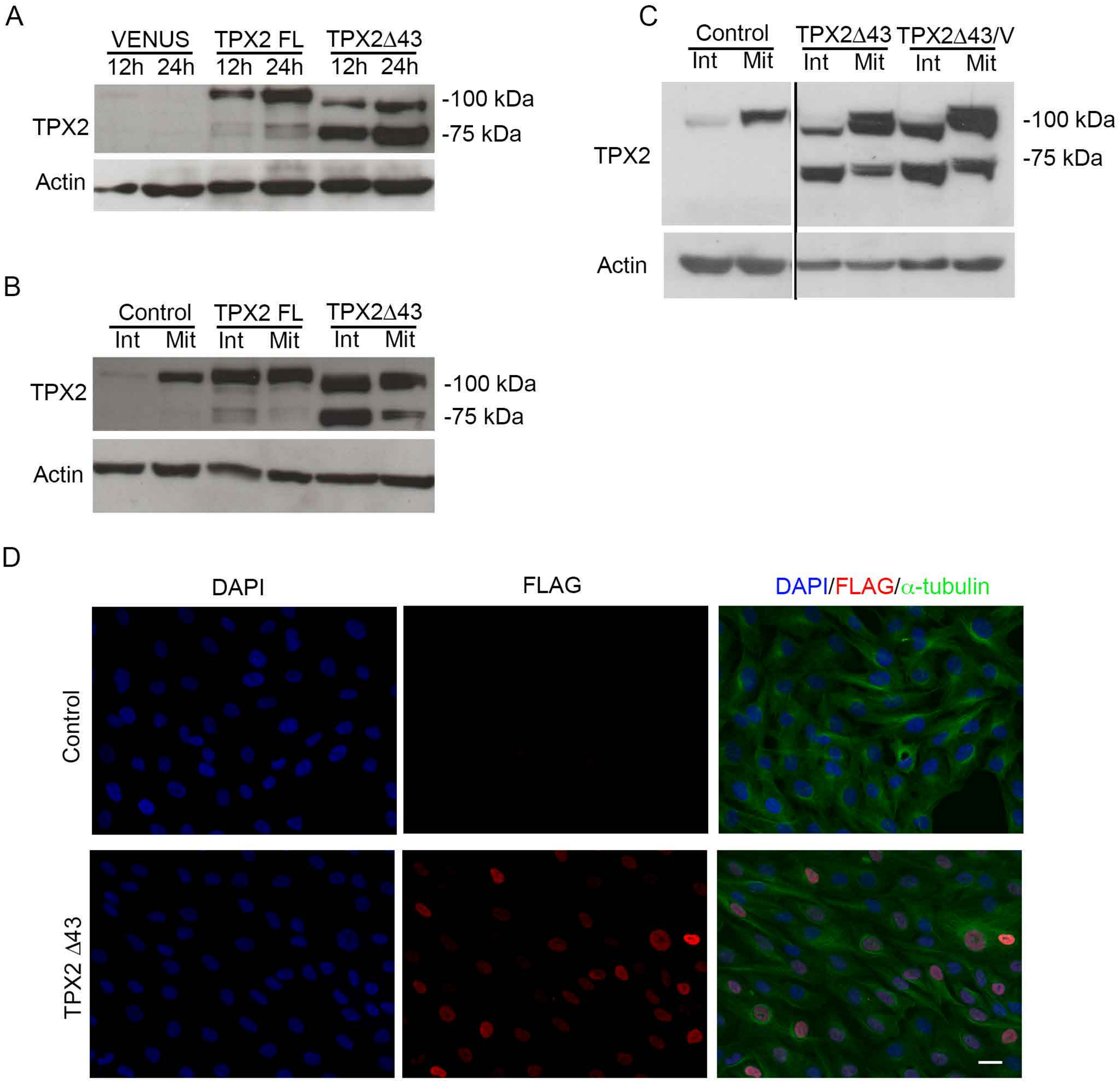

Supplementary Figure 2. TPX2Δ43 overexpression yields a further deleted N-terminal truncation. Western blot analyses show immunoblots performed on the same membranes of Figure 1, using the rabbit anti-TPX2 (Novus, NB500-179, 1:500) antibody in the indicated cell lines, after 12 and 24 h of doxycyclin induction (A), or in extracts from interphasic (Int) and mitotic (Mit) cells, 12 h after induction with doxycycline (B); a comparison of the cell lines co-overexpressing VENUS (TPX2Δ43/V) or not (TPX2Δ43) is shown in C. Actin is always used as loading control. D. Immunofluorescence panels show the inducible expression (+ dox) of FLAG-TPX2Δ43 after dox induction (12 h). Exogenous TPX2 displays the canonical localisation in interphase nuclei (visualised by DAPI staining). Scale bar: 25 µm.

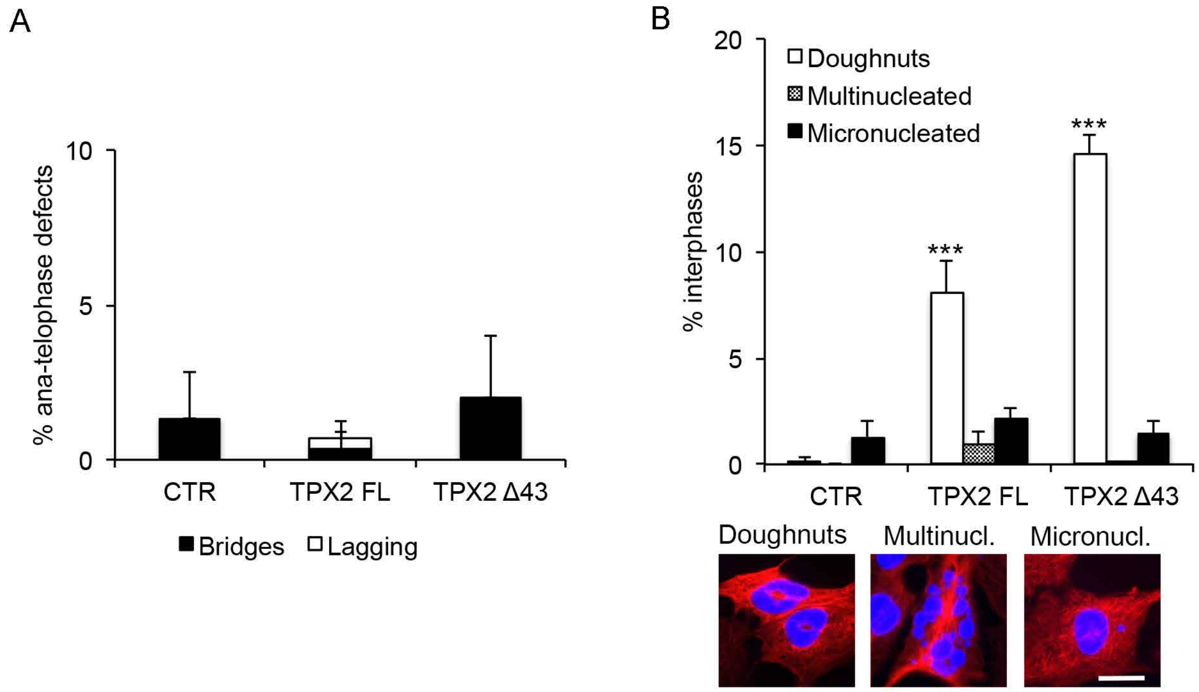

Supplementary Figure 3. Chromosome segregation defects in TPX2-overexpressing cells. Histograms display the percentage of chromosome segregation defects in ana-telophases (A) and interphasic nuclear defects (B) in the indicated cell lines in asynchronous growing cultures. Examples of interphasic phenotypes are represented in the IF panels. For histograms, at least 300 telophases (A) and1500 interphases (B) per condition were analysed, from 3 independent experiments; s.d. are shown; *** p<0.0001, chi-square (and Fisher's exact) test. Scale bar: 10 μm.

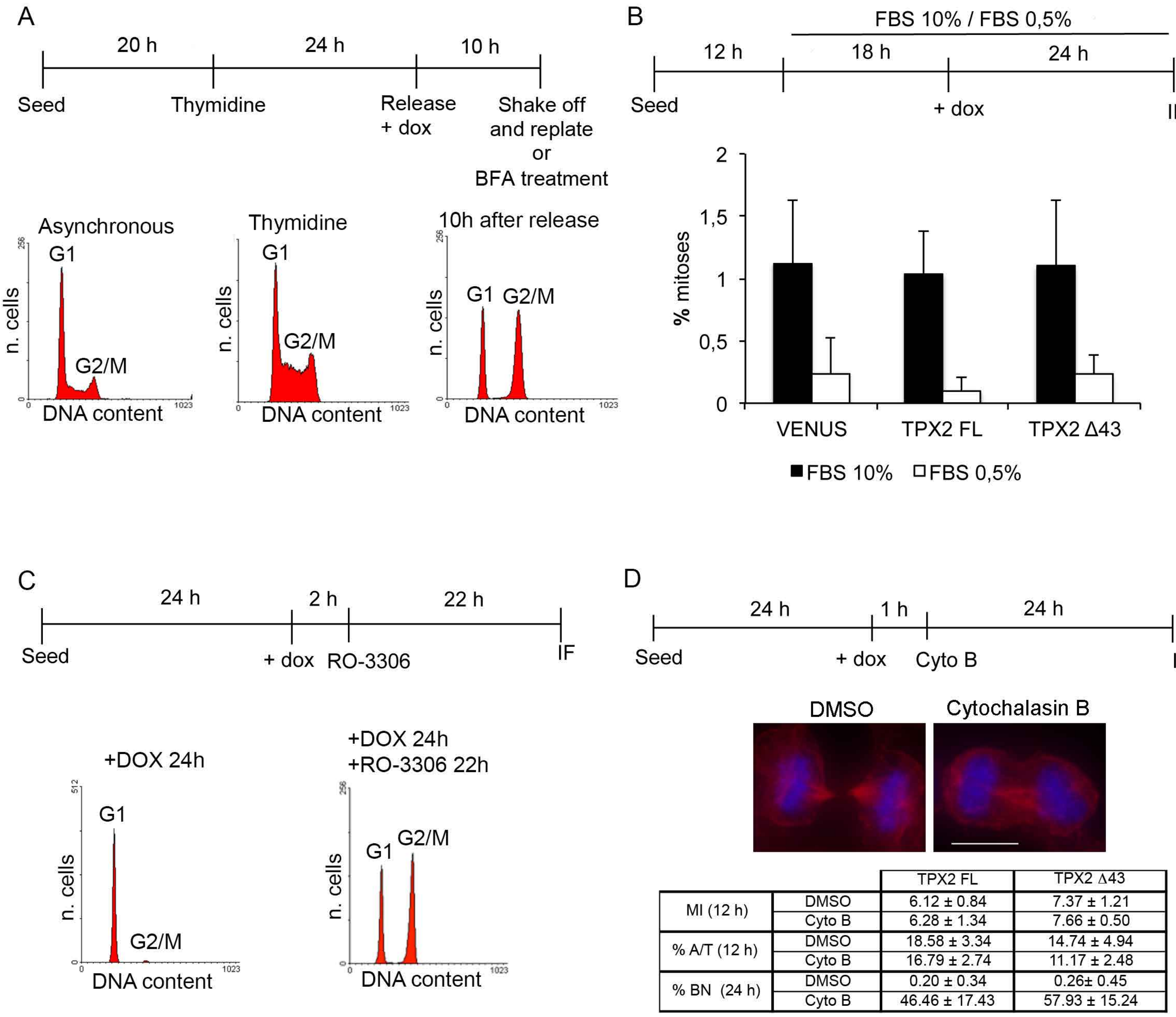

		TPX2 FL	TPX2 Δ43
MI (12 h)	DMSO	6.12 ± 0.84	7.37 ± 1.21
	Cyto B	6.28 ± 1.34	7.66 ± 0.50
% A/T (12 h)	DMSO	18.58 ± 3.34	14.74 ± 4.94
	Cyto B	16.79 ± 2.74	11.17 ± 2.48
% BN (24 h)	DMSO	0.20 ± 0.34	0.26± 0.45
	Cyto B	46.46 ± 17.43	57.93 ± 15.24

Supplementary Figure 4. Experimental protocols for synchronisation in different phases of the cell cycle and drug administration. Schematics of the experimental conditions used in the indicated cell lines to increase G2/M population (A), induce quiescence by serum starvation (B), enrich cultures in G2 cells by RO-3306 treatment , (C) or interfere with actin polymerisation (D). Time intervals are not represented to scale. As controls, we show the mitotic index (in A, at least 1500 counted cells from 3 different experiments; s.d. are shown), FACS profiles (B-C) and the intercellular bridge organisation (D). The mitotic index (MI; 1500 counted cells from 3 independent experiments), % of ana/early telophases (AT; 300 counted mitoses from 3 independent experiments) and of binucleated (BN; 1500 counted cells from 3 independent experiments) cells after cyto B treatment are shown in the Table in D (s.d. are indicated for all analyses). For FACS analyses, samples were permeabilised with PBS containing 0.1% TritonX-100. Cell cycle phase distribution was analysed after incubation with propidium iodide (0.04 mg/ml Sigma-Aldrich) using a flow cytofluorimeter Epics XL apparatus (Beckman Coulter). Scale bar: 10 μm.
